# Supplementary material for: Novel Eel Skin Fibroblast Cell Line: Bridging Adherent and Suspension Growth for Aquatic Applications Including Virus Susceptibility
Source: Biology (Basel). 2024 Dec 20;13(12):1068. doi: 10.3390/biology13121068 (PMC11673813; doi:10.3390/biology13121068)
Supplement: Supplementary file 1 [file biology-13-01068-s001.zip › Supplymentary Figure legend MDPI.pdf]

Figure S1. ES cells cultured at a low inoculum density. The growth curves of the passage 55 ES cells at a inoculum density of 2×10^5 cells·mL^{-1} (26 °C, 10% FBS). The values are exhibited as the mean ± standard deviation(SD), n = 3.

Figure S2. ES cells cultured under different FBS percentages (26 °C): the passage 55 ES cells with the FBS percentage of (**a**) 3%, (**b**) 5%, (**c**) 8%, (**d**) 10% and (**e**) 15% at 24 h, 72 h and 168 h post inoculation .

Figure S3. ES cells cultured under different temperatures (8% FBS). The passage 55 ES cells with the incubation temperature of (**a**) 15 °C, (**b**) 20 °C, (**c**) 26 °C, (**d**) 30 °C and (**e**) 37 °C at 12 h, 96 h and 168 h post inoculation.

Figure S4. PCR tests for the susceptibility of ES cells. (**a**) Detection of the AngHV DNA polymerase (*pol*) gene fragments. (**b**) The viral nervous necrosis (VNN) standard test fragment in the infected passage 54 ES cells following 3 subcultures.
