# Supplementary figures and images for: Novel Eel Skin Fibroblast Cell Line: Bridging Adherent and Suspension Growth for Aquatic Applications Including Virus Susceptibility
Source: Biology (Basel). 2024 Dec 20;13(12):1068. doi: 10.3390/biology13121068 (PMC11673813; doi:10.3390/biology13121068)

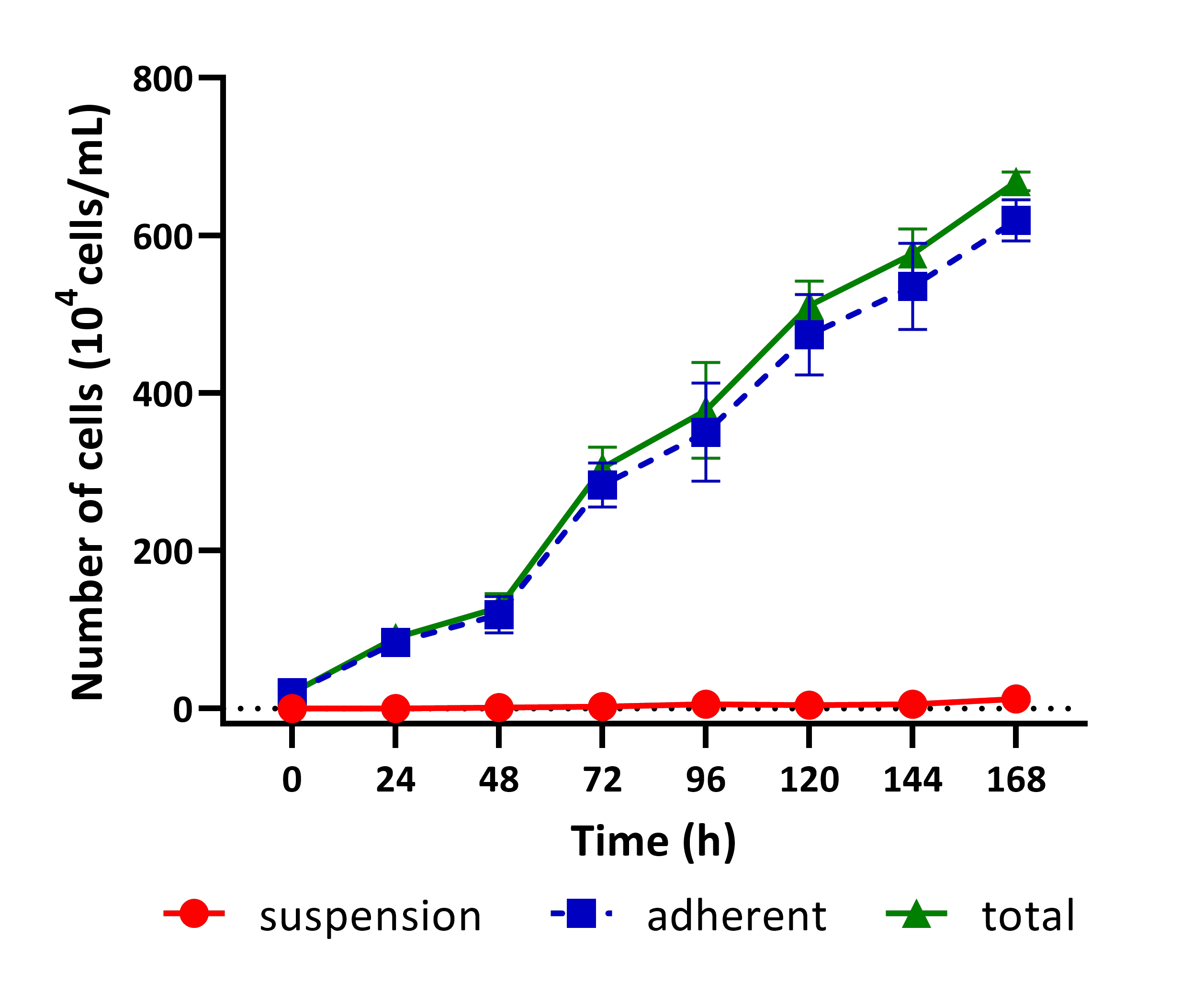

Supplement: Supplementary file 1 [file biology-13-01068-s001.zip › Figure S1.tif]

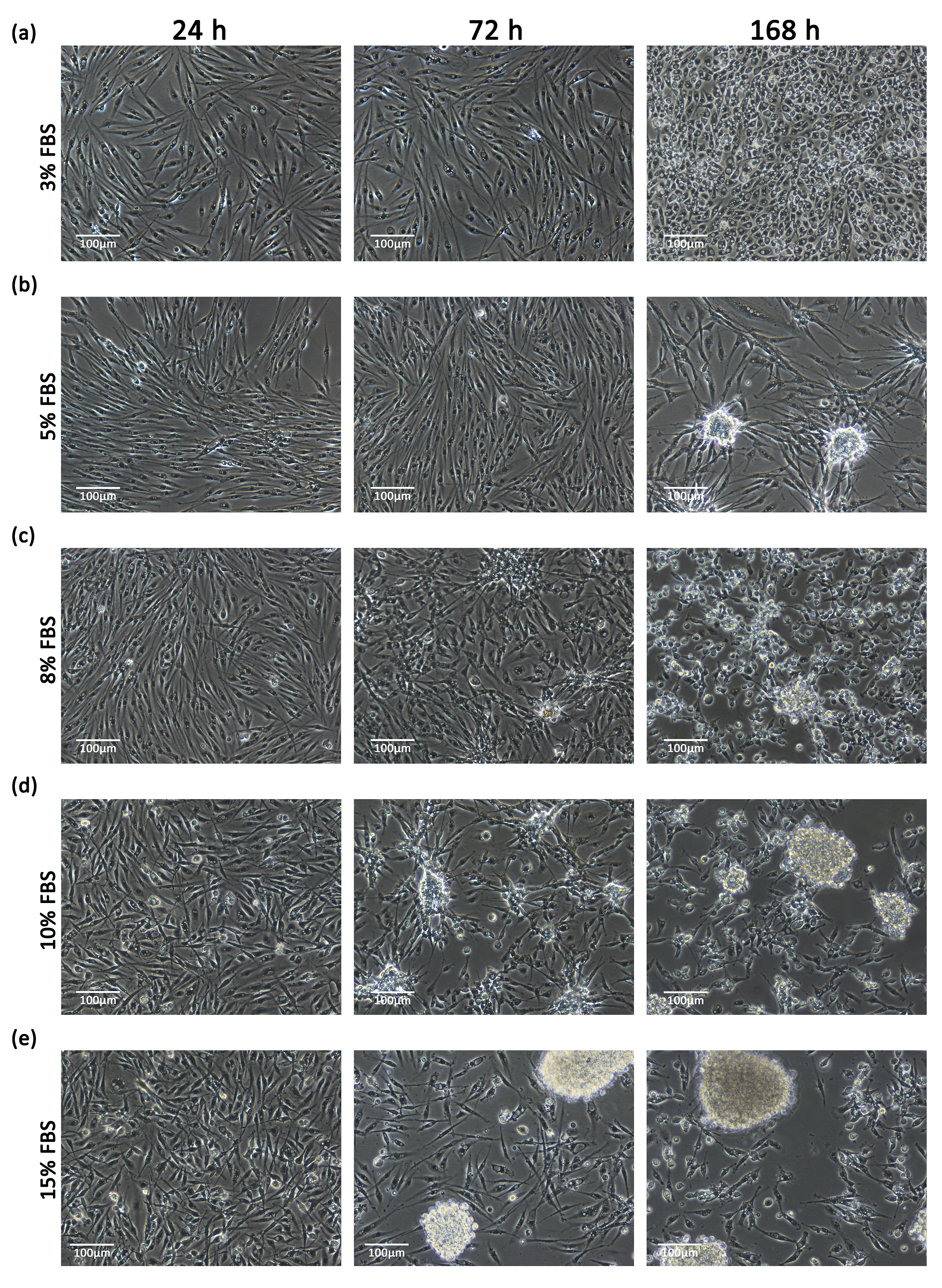

Supplement: Supplementary file 1 [file biology-13-01068-s001.zip › Figure S2.tif]

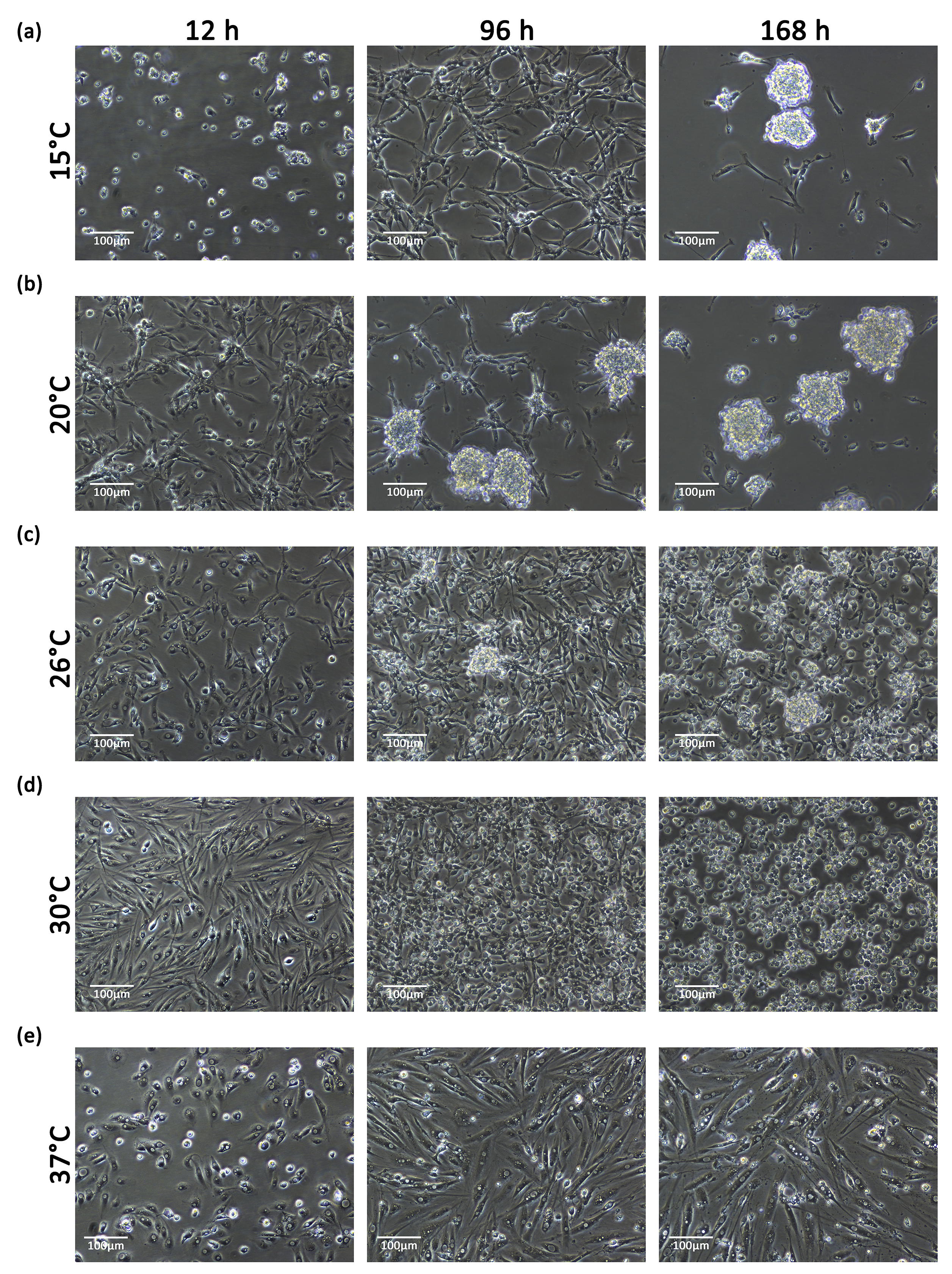

Supplement: Supplementary file 1 [file biology-13-01068-s001.zip › Figure S3.tif]

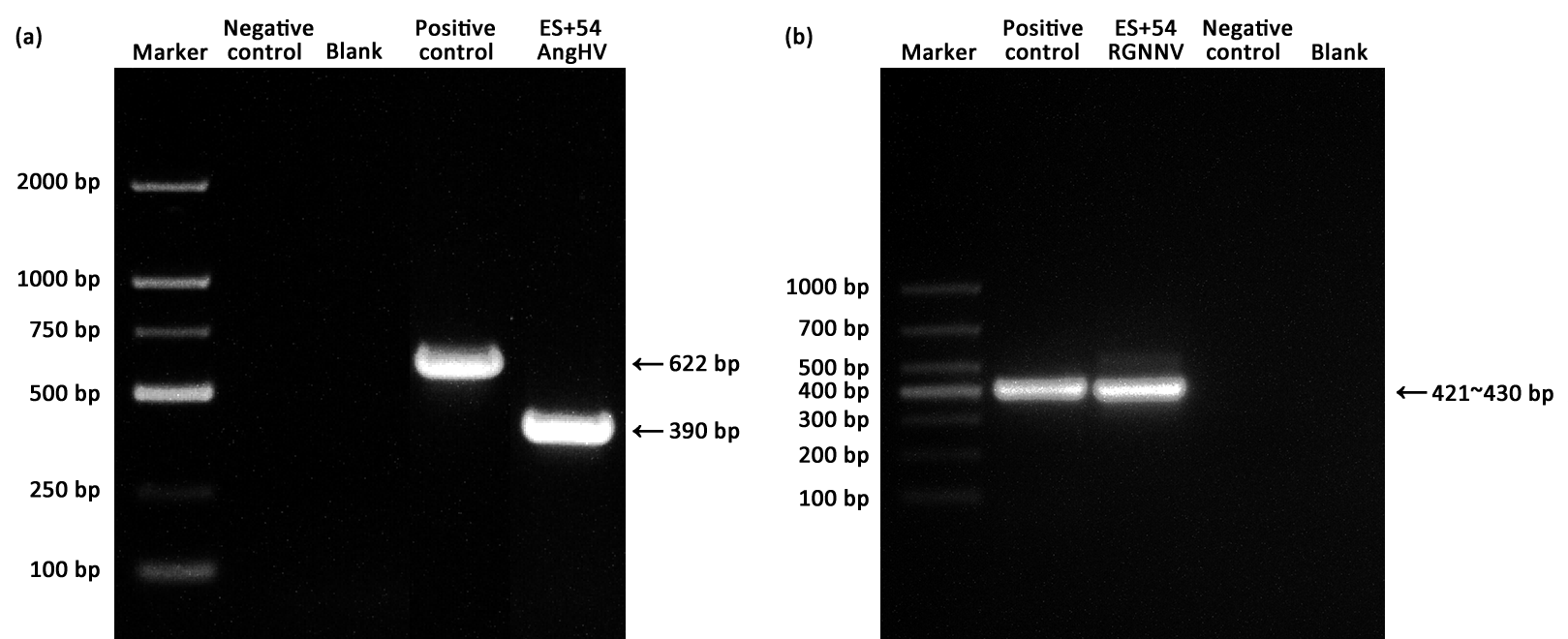

Supplement: Supplementary file 1 [file biology-13-01068-s001.zip › Figure S4.tif]
